# YK-4-279 Attenuates Progression of Pre-Existing Pigmented Lesions to Nodular Melanoma in a Mouse Model

**DOI:** 10.3390/cancers14010143

**Published:** 2021-12-29

**Authors:** Lee Huang, Yougang Zhai, Cristian D. Fajardo, Deborah Lang

**Affiliations:** Department of Dermatology, Boston University, Boston, MA 02118, USA; leehuang@bu.edu (L.H.); younzhai@gmail.com (Y.Z.); cfajardo@bu.edu (C.D.F.)

**Keywords:** melanoma, mouse models, ETS factors, small molecule inhibitors, therapeutics

## Abstract

**Simple Summary:**

Therapeutic options for melanoma are limited. In a prior study, we discovered the small molecule compound, YK-4-279, blocked tumor progression in a mouse model of melanoma. Tumor induction and drug administration occurred concurrently in this previous work. The aim of our current study was to test the efficacy of YK-4-279 in mice with already initiated but not progressed melanoma lesions. We have found that YK-4-279 was still able to attenuate melanoma progression significantly, although not to the degree as the prior trial. Using a preclinical in vivo mouse model that has relevancy to human disease, our findings support that there is promise for YK-4-279 as an option for melanoma therapy.

**Abstract:**

More options are needed for the effective treatment of melanoma. In a previous study, we discovered the small molecule drug YK-4-279 almost completely inhibited tumor progression in the *Braf^CA^*;T*yr-CreERT2*;*Pten^flox/flox^* transgenic mouse model. YK-4-279 had no effect on tumor initiation but blocked progression of invasive melanoma. Our current study was designed as a treatment model, where YK-4-279 was administered during pigmented lesion formation. The study design included the use of three groups: (1) a control group that received only DMSO without a drug (MOCK), (2) mice following our prior studies with YK-4-279 administered at the time of tumor induction (YK-4-279), and (3) mice treated during tumor initiation (YK-4-279 delay). While the MOCK mice had progression of tumors, both YK-4-279 and YK-4-279 delay groups had a significant block or delay of progression. The majority of mice in the YK-4-279 groups had a block of progression, while the YK-4-279 delay group had either a partial block (60% in male mice or 29% in females) or a delay in disease progression in females (28 days in controls to 50 days in YK-4-279 delay group). Here, we demonstrate that YK-4-279 has a significant impact on blocking or delaying tumor progression in a pre-clinical treatment model of melanoma.

## 1. Introduction

Melanoma is an aggressive disease that evolves to tolerate once-effective therapeutic treatments. For this reason, a toolbox of alternate strategies is needed to inhibit disease progression, reduce the negative impact of metastasis, or even cure this cancer type. We have previously reported the use of a small molecule inhibitor, YK-4-279, in a transgenic mouse model of melanoma [1]. The YK-4-279 compound was identified to inhibit ETS transcription factors, a protein family that includes 27 members [2,3,4]. One mechanism of how YK-4-279 inhibits ETS factor function is by blocking protein–protein interactions between multiple ETS proteins and binding partners RNA helicase A, p68 DDX5, and PAX3 [1,2,5]. We discovered that the majority of ETS family members were expressed in melanoma and that YK-4-279 inhibited the function of many of these ETS factors [1]. Using cultured cell model systems, we revealed that YK-4-279 blocked ETS factor interaction with the binding partner PAX3, attenuated ETS factor transcriptional function, and reduced the expression of the PAX3 and ETS1 downstream target gene MET. Due to the high number of ETS family members expressed and active in melanoma, and the number of pro-tumor activities these factors drive in this cancer type, it is logical that a compound that would inhibit these factors would negatively impact on melanoma progression. To test the impact of YK-4-279 treatment on tumor initiation and progression, a mouse model that mimics human disease (*Braf^CA^*;T*yr-CreERT2*;*Pten^flox/flox^* mice) was treated with YK-4-279. Indeed, we discovered that YK-4-279, while having no significant effect on tumor initiation, actively inhibited melanocytic lesions from progressing into invasive melanomas. Further, our in vivo outcomes paralleled the in cellulo findings, where YK-4-279 blocked ETS1 interaction with PAX3 and lead to a significant reduction of MET expression. Our findings support that YK-4-279 has potential as a melanoma therapeutic.

In our prior study, transgenic tumor induction and YK-4-279 treatment were performed at the same time. In the current study, to more closely mimic a clinical scenario, tumors were first induced and YK-4-279 was administered at the onset of tumor initiation and establishment, 13–21 days later. Here, we found that there was still significant delay or block of tumor progression but to a lesser degree than that of the earlier study. Further, we found differing outcomes based on the sex of the mouse. Coupled with our prior study, these findings are the first to examine YK-4-279 in a clinically relevant mouse model and provide evidence that the current clinical trials in Ewing sarcoma using the YK-4-279 chemical analog TK216 [6] may have similar promising effects in melanoma patients.

## 2. Materials and Methods

### 2.1. Melanoma Mouse Model

The transgenic mouse line*, Braf^CA^;Tyr-CreERT2;Pten^flox/flox^* mice, were obtained from Jackson Laboratories ([7], stock number 013590, C57BL/6J background). The transgenic mice possess a tamoxifen-inducible Cre expressed by a melanocyte-specific tyrosinase promoter (*TyrCreER*) that will activate a mutant oncogenic Braf allele (*Braf^CA/+^*), and delete expression of the tumor suppressor gene Pten (*Pten^flox/flox^*) after exposure to topical tamoxifen (4-hydroxy tamoxifen (4-OHT)) [7]. For breeding, mice were kept in double homozygous transgenic lines (*Braf^CA/CA^; Pten^flox/flox^ or Tyr-CreERT2^+/+^; Pten^flox/flox^*) and bred together to get triple transgenic experimental mice. These experimental mice (*Braf^CA^;Tyr-CreERT2;Pten^flox/flox^*) are heterozygous for *Braf^CA^* and T*yr-CreERT2* and homozygous for *Pten^flox/flox^*. This breeding strategy was followed due to some previously documented spontaneous tumor rates in breeding triple transgenic mice (as per Jackson laboratories). Genotyping was verified using primer sets and conditions as reported on the Jackson laboratory website. The genotyping primers for *Braf^CA^* are TGA GTA TTT TTG TGG CAA CTG C (forward) and CTC TGC TGG GAA AGC GGC (reverse) with amplicons of 308 base pairs for the transgenic allele and 185 base pairs for the wild type (Jackson laboratory protocol #29217). For T*yr-CreERT2*, a generic Cre genotyping protocol was followed, with forward primer GCG GTC TGG CAG TAA AAA CTA TC and reverse GTG AAA CAG CAT TGC TGT CAC TT, with internal control primers CTA GGC CAC AGA ATT GAA AGA TCT and GTA GGT GGA AAT TCT AGC ATC ATC C (Jackson laboratory protocol #22392). Resultant amplicons are 100 base pairs for the Cre transgene and 324 base pairs for the internal control primer set. For *Pten*, primers used were CAA GCA CTC TGC GAA CTG AG (forward) and AAG TTT TTG AAG GCA AGA TGC (reverse), with amplicon sizes 328 base pairs (floxed allele) and 156 base pairs (wild type allele) (Jackson laboratory protocol #233492). Mouse colonies at Boston University were maintained under specific pathogen-free conditions, and experimental procedures were performed in accordance with the approved protocols through the Boston University Institutional Animal Care and Use Committee (IACUC). For tumor induction, 20–50 microliters of 4mM tamoxifen (4-OHT, Sigma Aldrich, St. Louis, MO, USA) was applied to the shaved dorsal back skin of six- to seven-week-old *Braf^CA^;Tyr-CreERT2;Pten^flox/flox^* mice for three consecutive days (experimental days 1, 2, 3). Methods for determining tumor initiation (pigmented superficial lesion) and progression (raised nodular melanoma) were performed as previously described [8]. The recording of “tumor initiation” date was at the first sign of a pigmented lesion on the skin surface. The determination of the “tumor progression” day was when lesions formed a palpable mass. Mice were observed daily.

### 2.2. YK-4-279 Drug Treatment

To achieve steady systemic YK-4-279 levels (YK-4-279, Apexbio Technology #A3946), drugs were administered via osmotic mini-pumps (Alzet model 1004). Mice were assigned to one of three groups: a control group with pumps filled with DMSO only (MOCK group); mice following our prior study protocol [1] with 1.12 mM YK-4-279 total/final concentration in the pump, averaging 1.6 mg/Kg systemic drug levels (YK-4-279 group); and mice with a 5 times higher level of YK-4-279, with 5.6 mM total/final concentration leading to approximately 8 mg/Kg systemic drug levels (YK-4-279 delay). The first two groups (DMSO and YK-4-279) were surgically implanted concurrently with tamoxifen treatment, while the third group (YK-4-279 delay) was implanted 13–21 days after tumor induction and initiation. The use of osmotic mini-pumps is an alternative to repeated intraperitoneal injections of YK-4-279 over a long-time course. This strategy not only reduces handling stress to the mice, but also provides a mechanism for controlled and continuous delivery of the drug over the time course. For pump implantation, mice were anesthetized using isoflurane delivered through a nose cone, where responsiveness was tested through a toe pinch. Concurrent pre-emptive analgesics were administered during the procedure. The surgical area in the lower abdomen was shaved and sterilized with a triple treatment of ethanol and an iodine solution, and a 1 cm skin incision was made in the lower abdomen approximately half a centimeter to the right or left of the linea alba to get a good angle for insertion. The musculoperitoneal layer directly underneath was secured, and an incision into the peritoneal wall was created. Pumps were inserted into the peritoneal cavity with the delivery portal end first. The musculoperitoneal layer followed by skin layer was then closed with absorbable sutures, taking care to avoid disturbing or injuring the bowel. Pumps were maintained for no more than one half-life after the completion of the pump infusion time of 4 weeks to avoid reduced function following procedures outlined from the manufacturer. If necessary, a second minipump would be implanted at day 28 or 29. Osmotic minipumps have a release rate of drug of 0.11 uL/hour. Mice were removed from the study when tumor burden exceeded 1cm^2^ or between day 65 and 85 if there was no progression detected.

### 2.3. Statistical Analysis 

Statistical tests were performed using GraphPad Prism6 (GraphPad Software Inc., La Jolla, CA, USA). Kaplan–Meier survival plots were generated on Prism6 and analyzed using Log-rank (Mantel-Cox) tests with one degree of freedom. Sample sizes were calculated using a power analysis, with power > 85% for mouse experiments. Findings are presented as significant if the p values were less than or equal to 0.05.

## 3. Results

**Figure 1:**
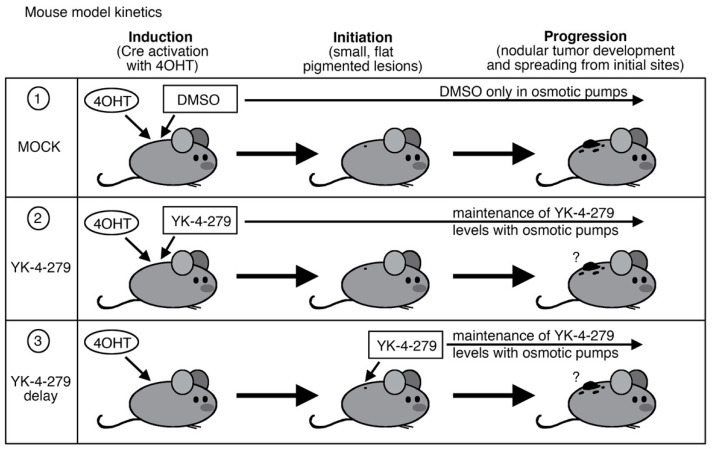
Schematic for study design. For all treatment groups, mice were induced with 4-hydroxy tamoxifen (4-OHT) on day one of the study. Row (**1**), MOCK control group, was treated with DMSO; row (**2**), YK-4-279 group, was treated with YK-4-279 on day 1; and row (**3**), YK-4-279 delay group, was treated two weeks after induction and initiation. For each group, mice had osmotic pumps surgically implanted in the intraperitoneal space with the drug or DMSO. For all mice, disease benchmarks of the first observation of a pigmented lesion (initiation) and progression to a palpable lesion (progression) were recorded.

**Figure 2:**
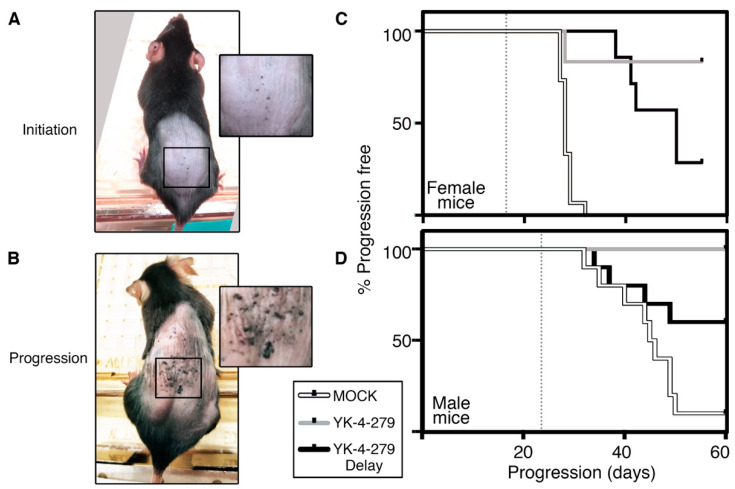
YK-4-279 inhibits melanoma progression in a mouse model of melanoma. (**A**,**B**) Gross morphology of mice with initiation (**A**) and progression (**B**) lesions. Boxed area indicated is magnified 200% in insets. (**C**,**D**) Kaplan–Meier progression free survival graphs of female (**C**) and male (**D**) mice. Groups on each graph are indicated as MOCK groups (white line), YK-4-279 groups (grey lines), and YK-4-279 delay groups (black lines). Dotted grey lines mark the average (median) days for tumor initiation for female and male MOCK groups. Both male and female YK-4-279 and YK-4-279 delay groups are significantly different from MOCK groups (*p* < 0.0001 male and female YK-4-279 groups and female mock delay, *p* = 0.047 male YK-4-279 delay). Mouse numbers for MOCK, YK-4-279, and YK-4-279 delay were *n* = 15, 12, and 7 (females) and *n* = 10, 11, and 10 (males), respectively. The study has at least 85% power for each comparison of the two YK-4-279 groups to sex matched MOCK controls.

In our prior report regarding melanoma [1], YK-4-279 was tested to determine if this compound could inhibit tumor initiation or progression. The *Braf^CA^;Tyr-CreERT2;* *Pten^flox/flox^* transgenic mouse was utilized as a melanoma model (Jackson Laboratory stock number 013590, C57BL/6) using procedures in accordance with the Institutional Animal Care and Use Committee (IACUC) approved protocols. This model was selected due to similarities with human melanoma pathology, including genes involved, kinetics, and histology [7,9]. In our prior report, mice were (1) induced on study day 1 with 4 mmol/L 4-hydroxy-tamoxifen (4-OHT, Sigma Aldrich) and (2) transplanted with osmotic pumps (Alzet model 1004) filled with either YK-4-279 (releasing drug at 1.6 mg/Kg, Apexbio Technology #A3946) or DMSO alone, shown schematically in Figure 1 row 1 (MOCK) and row 2 (YK-4-279). In these prior studies, a significant sex bias occurs in this melanoma model [8], so males and females were evaluated separately. Mice were evaluated at two time points: initiation as indicated with the first sighting of a pigmented lesion (Figure 2A) and progression where the precursor lesions evolved into palpable lesions (Figure 2B) (described in detail in [8]). From our prior studies shown here [1], no significant differences in tumor initiation times were detected. In control experiments, initiation occurred at 17.9 ± 2.3 days in female mice, and 22.7 ± 3.6 days in males, with no statistically significant difference in tumor initiation after treatment with YK-4-279 treatment (18.3 ± 1.3 days in females and 22.1 ± 1.9 days in males [1]). In addition, the incidence curves were not significantly different (X^2^ = 0.03, *p* = 0.86 females, X^2^ = 0.58, *p* = 0.45 males, log rank tests). However, in our prior and current studies, we found a significant impact on progression of the initiated pigmented lesions to nodular tumors. In female mice, all control mice progressed before or by day 32, but only 2 out of 12 mice progressed with YK-4-279 treatment (Figure 2C, white (MOCK) and grey (YK-4-279) lines), resulting in a significant inhibition of progression (X^2^ = 18.8, *p* < 0.0001, log rank tests, 1df). These findings were replicated in the male mice, with 90% (9 out of 10) of the control group having progressive melanoma but none in the YK-4-279 treated group (Figure 2D, white (MOCK) and grey (YK-4-279) lines, X^2^ = 18.25, *p* < 0.0001, log rank tests, 1df). In our prior studies, we found a significant block in melanoma progression in our mouse models when YK-4-279 was administered during tumor induction.

To test the efficacy of YK-4-279 as a therapeutic, we delayed the administration of YK-4-279 until after tumor induction, at the onset of tumor initiation, and establishment (Figure 1 row 3, YK-4-279 delay). For these studies, two major changes were made from our previous work. First, rather than induce tumorigenesis with 4-OHT and implant osmotic pumps on day 1, pumps were transplanted when pre-cancerous pigmented lesions arose in the model at 13 to 21 days. Second, YK-4-279 levels were increased five-fold, to 8 mg/Kg. Even with this increase, our levels were still well within the range for what can be given clinically and well below previous studies that used daily injections at 75–150 mg/Kg [2,4]. We maintained the use of osmotic pumps since this method provided the advantage of constant and consistent drug levels, as well as less stress and handling of the mice. Further, this method avoided the relatively quick systemic clearance of YK-4-279, which was remedied through the use of the controlled drug release of the osmotic pump [10,11]. These additional studies (Figure 1 row 3) were performed concurrently or overlapping with our prior reported experiments (Figure 1 rows 1,2).

Following the modified method for the YK-4-279 delayed group, there was again a significant impact towards delaying or inhibiting tumor progression. In female mice, the timing of the progression in the YK-4-279 delay group was significantly slowed when compared to the MOCK control group (Figure 2C, black line (YK-4-279 delay) versus MOCK control (white line)). The differences in the survival curves were significant using Log Rank/Mantel–Cox tests (X^2^ = 18.09, *p* < 0.0001, log rank tests, 1df). While the majority of female mice in the YK-4-279 delay group progressed (71% compared to 100% in MOCK group and 17% in YK-4-279 group) there was a significant increase in median progression free survival time, from 28 days in the MOCK group to 50 days in the YK-4-279 delay group. In the male mice, there was also a significant increase in progression free survival time (Figure 2D, black line (YK-4-279 delay) versus MOCK control (white line)), using Log-rank/Mantel–Cox tests (X^2^ = 3.93, *p* = 0.0474, log rank tests, 1df). At the end of the study, 60% of the male mice were progression free (compared to 10% in the MOCK group and 100% in the YK-4-279 group). While there was an overall increased significant progression-free survival of the males in the YK-4-279 delay group when compared to controls, there was not a significant difference in earlier time points with overlap of the two curves. This was supported when Gehan–Breslow–Wilcoxon tests were utilized, where more weight was given to earlier time points (X^2^ = 2.52, *p* = 0.1124). This differed from the significant differences between curves for the male MOCK group and YK-4-279 group (X^2^ = 16.37, *p* < 0.0001) and both YK-4-279 female groups compared to controls (YK-4-279 X^2^ = 14.37, *p* = 0.0002, YK-4-279 delay X^2^ = 13.87, *p* = 0.0002). At the end point of the study, 2/7 of females and 6/10 of males were progression-free in the YK-4-279 delay group. Since this model is prone to develop secondary non-pigmented tumors [9] all mice that were still progression-free were collected at day 52–60 for the female mice and days 65–85 for the male mice.

In summary, YK-4-279 had an impactful outcome on melanoma progression in the *Braf^CA^;Tyr-CreERT2;Pten^flox/flox^* mouse model when treatment started at the onset of tumor initiation (YK-4-279 delay group). A subset of mice in this group (60% of males and 29% of females) did have a block of tumor progression. While the female mice had a lower level of progression-free survival than the male mice, all the female mice had a delay of the onset of progression. In contrast, the YK-4-279 delay group males either fully responded to the drug or had disease progression at a similar rate to the MOCK control group. This study demonstrates that YK-4-279 has potential as a melanoma therapeutic and that sex differences should be considered.

## 4. Discussion

Our findings here demonstrate the potential for YK-4-279 as a therapeutic for melanoma. Initially, YK-4-279 was identified as an inhibitor of the ETS-factor containing EWS-FLI1 but was later discovered to affect other ETS factors, such as ETS1, ETV5, ERG, and ETV1 [1,2,3,4]. However, our work and others support that YK-4-279 has pleiotropic effects, including disruption of the mitotic spindle assembly and polymerization [1,12,13]. Regardless of the off-target pleiotropic effects, our findings are promising with the significant attenuation of tumor progression of established melanoma lesions in our mouse model. Due to the potential of this compound, a clinical derivative of YK-4-279 (TK216) is currently in clinical trials for relapse or refractory Ewing sarcoma patients with early encouraging findings [6]. In our prior study, where treatment and induction of tumor occurred concurrently, YK-4-279 treatment was able to block progression in the majority of mice [1]. However, when YK-4-279 was administered after the establishment of a pigmented lesion, there was only a partial rescue from tumor progression (Figure 2C,D).

In this study, we found that YK-4-279 had a significant negative impact on cancer progression. However, these studies can be expanded and improved. Firstly, in these experiments, all treatment groups were compared to mock-treated mice where the pumps were transplanted concurrently with tumor induction. It can be argued that a second control, where DMSO-containing pumps are inserted at days 13–21 in parallel with our YK-4-279 delay group treatment, should also be included. Further, we focused on young mice, and the inclusion of older mice would also be informative since age is positively correlated with incidence for melanoma and other cancers [14]. In addition, our model is driven by an activating mutation in BRAF, which is the most common genetic defect found in melanoma, occurring in about half the cases. However, a significant number of patients (approximately 20%) have mutations in NRAS, which occur almost mutually exclusive of BRAF mutations [15]. While both BRAF and NRAS are members of the MAPK pathway, melanoma tumors with these different mutations have divergences in terms of tumor aggressiveness, drug response, age of onset, and correlation with melanoma subtype [16,17]. For a more comprehensive study, this work should be expanded to include other mouse models of melanoma that are driven by NRAS or other mutations. Considering that several ETS factors are direct targets of MAPK pathway signaling [18,19], an examination of YK-4-279 treatment for both BRAF and NRAS driven melanomas has significance.

One strategy to improve progression-free survival in our model is to use YK-4-279 (or the clinical derivative TK216) as a combination therapeutic. No studies using YK-4-279/TK216 as a combination therapy in melanoma are currently reported, and it is unknown if a combinational therapy approach would have any impact, either additively or synergistically. To identify combinational drug partners to expand on YK-4-279 anti-cancer effects in Ewing sarcoma, Zollner and colleagues performed a multi-drug screen and revealed several tubulin modulators and microtubule inhibitors that included vinca alkaloids but not taxanes [20]. Use of the alkaloid compound vincristine demonstrated enhanced growth arrest and cytotoxicity in both Ewing sarcoma cell culture and mouse xenograft models when combined with YK-4-279. Currently, vincristine combination therapy was added to the on-going clinical trial for Ewing sarcoma with promising early findings [6]. It is unknown if YK-4-279 or the TK216 derivative vincristine combinatorial impact with Ewing sarcoma tumor progression will translate to another tumor type such as melanoma. An alternative to the use of vincristine is to utilize current melanoma treatment options as combination therapeutics, for example immunotherapeutics (such as anti-PD-1 targeting drugs) or compounds that target BRAF V600E mutations [21]. However, there is good reason for optimism that the use of YK-4-279/TK216 in melanoma patients will mirror the findings in Ewing sarcoma since preliminary results in other cancer types, such as prostate, thyroid cancer, leukemia, and various forms of lymphoma, have shown similar promising results [4,11,22,23].

The *Braf^CA^*; T*yr-CreERT2*;*Pten^flox/flox^* transgenic mouse model tested in this study has a significant sex bias in terms of tumor kinetics of initiation and progression [8]. When YK-4-279 was administered at induction, the sex bias difference for initiation was evident, but there was significant block of progression in both sexes [1]. In the current study, there is still a significant sex bias in the response to the drug. In female mice, the progression of disease is significantly delayed, but eventually, the majority (71%) had tumor progression (Figure 2C). In males, the mice had one of two outcomes: (1) progression-free survival during the duration of the study (60%) or (2) progression without significant differences from the MOCK control group (Figure 2D). It is not clear if this sex difference in response to YK-4-279 is due to the mouse model itself and/or melanoma. One consideration is in the design of the model and the use of tamoxifen; therefore, sex differences may be due to the function of tamoxifen as an estrogen receptor modulator that may have differential effects in male and female mice. There are also findings of sex differences in melanoma incidence in patients. For example, in younger patients (under 45 years old) there is a higher incidence of disease in women, but this significantly switches to a male bias in older patients coupled with higher mortality rates [24,25,26,27].

## 5. Conclusions

In summary, we find a partial but significant attenuation of melanoma progression in *Braf^CA^*; T*yr-CreERT2*;*Pten^flox/flox^* transgenic mice using YK-4-279 as a single agent. The ability of YK-4-279 to possess anticancer effects across a variety of cancer types illustrates its promise as a therapeutic option, and our discovery of this compound’s effectiveness in a clinically relevant, post-tumor-induced mouse model further highlights this potential. Pan-inhibition of a family of oncogenic transcription factors, such as ETS, demonstrates promise for drug targeting but may leave room for improvement. Due to the incomplete protection against tumor progression in the tested melanoma model system, a strategy that incorporates a combination with other therapeutics will further enhance the anti-tumor effects of YK-4-279.

## Data Availability

The data presented in this study are available on request from the corresponding author.
